# Psychological and Physiological Responses following Repeated Peer Death

**DOI:** 10.1371/journal.pone.0075881

**Published:** 2013-09-27

**Authors:** Judith Pizarro Andersen, Roxane Cohen Silver, Brandon Stewart, Billie Koperwas, Clemens Kirschbaum

**Affiliations:** 1 Department of Psychology, University of Toronto Mississauga, Mississauga, Ontario, Canada; 2 Department of Psychology and Social Behavior, University of California Irvine, Irvine, California, United States of America; 3 Department of Government, Harvard University, Cambridge, Massachusetts, United States of America; 4 Department of Psychology, Cornell University, Ithaca, New York, United States of America; 5 Department of Psychology, Technische Universitat Dresden, Dresden, Germany; Federal University of Rio de Janeiro, Brazil

## Abstract

**Objective:**

Undergraduates at a university in the United States were exposed – directly and indirectly – to 14 peer deaths during one academic year. We examined how individual and social factors were associated with psychological (e.g., anxiety, depression, somatization) and physiological (i.e., cortisol) distress responses following this unexpected and repeated experience with loss.

**Method:**

Two to three months after the final peer death, respondents (N = 122, 61% female, 18–23 years, M = 20.13, SD = 1.14) reported prior adverse experiences, degree of closeness with the deceased, acute responses to the peer deaths, ongoing distress responses, social support, support seeking, and media viewing. A subset (n = 24) returned hair samples for evaluation of cortisol responses during the previous 3 months.

**Results:**

Ongoing psychological distress was associated with a) prior interpersonal trauma, b) fewer social supports, and c) media exposure to news of the deaths (*p's*<.05). Participants who had no prior bereavements showed, on average, high cortisol (>25 p/mg) compared to individuals with one or two prior bereavement experiences (who were, on average, within the normal range, 10 to 25 p/mg) (*p*<.05). Only 8% of the sample utilized available university psychological or physical health resources and support groups.

**Conclusions:**

Limited research has examined the psychological and physiological impact of exposure to chronic, repeated peer loss, despite the fact that there are groups of individuals (e.g., police, military soldiers) that routinely face such exposures. Prior adversity appears to play a role in shaping psychological and physiological responses to repeated loss. This topic warrants further research given the health implications of repeated loss for individuals in high-risk occupations and university settings.

## Introduction

Mass violence and collective losses such as school shootings and terrorist attacks have been associated with lingering psychological and physical health effects for individuals both directly and indirectly exposed [1], [2]. We examined the impact of a community tragedy that occurred during one academic year at a university in the United States. In just over 9 months, 14 students died from suicide, illness, and accidents. Unlike collective traumas occurring at one point in time, students at this university were exposed to repeated losses over an extended period of time, within which large clusters of deaths (particularly the 8 suicides) happened in rapid succession, some within the same week. Although some students knew personally one or more of the individuals who died, most were only indirectly exposed to the loss of their peers, learning about the deaths from friends or media. There is an absence of research examining the psychological and physiological impact of direct and indirect exposure to an extended period of repeated peer loss, despite the fact that there are groups of individuals (e.g., police, military soldiers, fire fighters) that routinely face such exposures.

Factors that may exacerbate or mitigate psychological and physiological distress responses in the aftermath of traumatic events have been identified [1], [3], although it is unknown how these factors are associated with mental and physical health during and following extended periods of repeated peer loss. For example, existing literature suggests prior adversity may alter responses to subsequent adverse experiences such as collective loss. Some research supports an inoculation effect, whereas prior exposure to traumatic events appears protective against distress following future events. Norris and Murrell [4] argue that because traumatic events extend beyond normal human experiences, once an individual has experienced a particular traumatic event, future exposure should reduce the likelihood that such an event would be perceived as distressing. They found support for their hypothesis when studying victims of a flood disaster. Older adults who had no prior flood experience showed increased anxiety and distress responses to flood warnings compared to individuals who had previously experienced flooding. Similarly, Bornstein and colleagues [5] found that widows and widowers who had previous experience with the death of a relative reported lower levels of depression following their spouse's death than those without prior experience of losing a loved one.

In contrast, some researchers have found that repeated exposure to traumatic events can sensitize individuals, creating vulnerability to enhanced psychological and physiological distress following future adversity [6], [7]. For example, researchers examined the medical records and interview responses from over 17,000 patients of a large health organization and found a graded relationship between the number of prior childhood adversities (e.g., physical, sexual abuse) and an increased risk for problems across six domains in adulthood (i.e., affective, somatic, substance abuse, memory, sexual, and aggression) [6]. Dougall and colleagues [8] interviewed emergency workers responsible for cleaning up after a large airline disaster and collected physiological (i.e., heart rate, blood pressure and urinary catecholamine) data from the majority. Individuals who had experienced dissimilar prior adverse experiences (e.g., threat, assault) were more vulnerable to psychological distress than individuals who had experienced a similar trauma in the past (e.g., viewing or handling dead bodies), although no differences were seen in physiological arousal. Some have argued that adverse experiences can also alter Hypothalamic-Pituitary-Adrenal (HPA) (i.e. cortisol) responses to future stress [9], [10]. For example, in a small community sample, Resnick and colleagues [10] found physiological (i.e., cortisol) differences among women who had been raped, based on their prior trauma experience. Women who had never experienced a previous assault showed high cortisol levels and women who had experienced a previous assault showed attenuated cortisol responses, but were more likely to develop PTSD. There is also evidence that the number of prior adverse events matter. Seery and colleagues [11] conducted a longitudinal study on a national sample exposed to a collective trauma and found that individuals with a history of *no* (0) or a *high* (5+) number of adverse events reported higher global distress, more post traumatic stress symptoms, greater functional impairment, and lower life satisfaction compared to individuals with *some* (1–2) prior adversities.

A second line of research has identified social factors, such as closeness to deceased peers and availability of social support, that play a role in mental and physical health responses to collective loss. For example, results from a study of students indirectly exposed to the September 11^th^ attacks who had not experienced personal bereavement related to the attacks found that disaster-related distress was higher among students who identified with the victims and those who experienced lack of support from close relationships [12]. Hughes and colleagues [1] conducted a study examining exposures that were most predictive of PTSD in survivors of the Virginia Tech mass shootings and found that the inability to confirm the safety of friends during the event and deaths of both close and non-close friends most strongly predicted the onset of PTSD. Grills-Taquechel and colleagues [13] found that the perception of social support before the traumatic event was associated with lower self-reported anxiety and greater quality of life among female students in the months following the loss of peers during the Virginia Tech mass shooting. In particular, family and environmental support (e.g., access to tangible university resources, such as transportation and safety) were important social resources.

Finally, exposure to media reports about collective loss has also been associated with negative psychological and physical reactions. Pfefferbaum and colleagues [14] found a relationship between television exposure to the Oklahoma City bombing and distress symptoms among children. Further, in a nationally representative sample of U.S. adults, researchers found that early and frequent exposure to 9/11 and Iraq war images viewed indirectly (e.g., via television) were associated with post-traumatic stress symptoms and increased incidents of physical health ailments two to three years following the events [2].

In the present study, we examined the psychological and physical health effects of exposure to repeated loss among university students in the months following the cluster of peer deaths. We extended prior literature on the impact of exposure to collective trauma in several ways. We investigated a new area of research, the cumulative effects of repeated peer loss. We examined how individual (e.g., prior interpersonal trauma and bereavement experiences) and social (e.g., social support) factors were associated with psychological (e.g., anxiety, depression, somatization) and physiological distress responses following repeated loss events. We chose to examine physiological responses using hair cortisol, a retrospective marker of HPA activity [15]. Hair cortisol analysis is a relatively new technique that represents longer-term patterns and possibly trait stress HPA profiles rather than acute arousal states [15]. Given that the losses we examined were experienced over several months, hair cortisol served as a promising biomarker for measuring the physiological impact of cumulative loss over time.

We expected that the number and type (e.g., bereavement) of prior adverse experiences would be associated with the severity of acute responses, ongoing psychological distress, and elevated cortisol responses. Given the similarity between these university students and the peers who died, we expected that knowing personally more of the deceased peers and exposure to media reports of the deaths would be associated with the severity of acute responses, ongoing psychological distress, and elevated cortisol levels. We also expected that low levels of social support would be related to elevated distress responses.

## Methods

### Procedures

Recruitment emails with a link to an anonymous 30-minute online survey were sent to a random selection of all students (n = 1000) enrolled in a class with one of the deceased at the time of their deaths (email addresses provided by the university registrar). Emails were also sent to members of student organizations, sports teams, and friend groups of the deceased students (n = 67). Students created a personal identification code upon survey registration that was associated with their data. On survey completion, participants were directed to a separate secure website where they recorded their contact information and, if they chose to, signed up for a drawing for one of 12 gift cards offered ($20 each). The online surveys were available for completion during an 8-week period in the summer after the academic year of the peer deaths.

All students who provided contact information were subsequently sent hair sample collection kits (i.e., hair clip, tin foil wrapper for the hair storage) and a postage-paid return envelope with a space to provide their survey ID but no identifying information. Hair samples were to be cut close to the scalp from a posterior vertex position. A minimum of 50 mg of hair was obtained per participant. Upon return receipt of the hair kit, the personal ID from the survey was matched to the hair sample for data analysis. All procedures were approved by the Human Subjects Ethics Committee at Cornell University.

### Survey Measures

#### Demographics

Age, gender, race/ethnicity, and year in school were collected.

#### Relationship to deceased peers

Information about the participant's relationship to peer(s) who died was collected via a series of questions, rated on 5 point Likert scales (*Not at all* to *Much more than other students*) (e.g., “How many of the 14 did you know generally, as acquaintances?”; “How many did you know personally?”).

#### Media exposure to the deaths

Students reported how many hours, on average, they spent reading newspapers, web articles, listening to the radio or watching TV about the events in the first week after the deaths.

#### Social support and support-seeking

Students were asked how many individuals they could turn to for emotional support. Additionally, students were asked if, during the period of peer loss, they sought help to “deal with any physical or mental health symptoms they experienced related to any of the deaths” from several possible sources (psychiatrist, psychologist or counselor, primary care provider, other health care professional, campus crisis or other support hotline, peer support group).

#### Mental health history

Students were asked if they had ever been diagnosed with an anxiety disorder or depression prior to the first peer loss.

#### Prior adverse experiences

Lifetime exposure to negative life events was assessed using a measure that has previously been used to collect data from national samples [11]. Participants reported whether they had ever experienced one or more of a list of 37 adverse events. Based on definitions within clinical literature on traumatic events and ratings by a clinician specializing in trauma theory, adverse experiences were grouped into 3 categories: a) *interpersonal trauma* (e.g., physical or sexual assault), b) *bereavement events* (e.g., death of family member or friend other than a current peer loss); c) *other adverse events* (e.g., serious illness or injury to self, serious financial difficulty, experienced a natural disaster).

#### Distress responses

Participants were asked to report which of the 14 deceased students' deaths impacted them the most, what month this occurred, and the severity of their acute reaction to the news, from *no reaction* (0) to *extremely strong reaction* (5).

Using the Brief Symptom Inventory 18 (BSI-18) [16], respondents rated the degree to which symptoms of depression, anxiety and somatization had distressed or bothered them during each of the 12 months starting from the first death at the start of the of the academic year using 5-point Likert Scales (*Not at All* to *Extremely*). We also assessed respondents' current month distress levels. This was used to represent a level of *ongoing distress*.

#### Hair cortisol

Hair grows at approximately one centimeter per month, and like rings on a tree, monthly cortisol values can be read retrospectively [17]. Prior 3-month samples have been used as an objective indicator of chronic stress due to the observable disruption of normal function in the HPA axis [18]. The Davenport protocol was employed for washing hair and steroid extraction [19]. In brief, each hair segment was put into a 15 ml Falcon tube, then 2.5 ml isopropanol was added, and the tube gently mixed on an overhead rotator for three minutes. The hair samples were allowed to dry for at least 12 hours. Next, the hair segments were powdered using a Retsch ball mill (5 min at 30 Hz). Fifty milligrams of powered hair was weighed out and transferred into a 2 ml cryo vial (Eppendorf, Hamburg, Germany). Then, 1.5 ml of pure methanol was added and the vials then slowly rotated over 24 hours for steroid extraction. Samples were spun in a microcentrifuge at 10.000 rpm for 2 min, and 1 ml of the clear supernatant was transferred into a new 2 ml cryo vial. The alcohol was evaporated at 60 degrees Celsius under a constant stream of nitrogen until the samples were completely dried (duration: approx. 20 min). Finally, 0.4 ml of phosphate buffer was added and the tube vortexed for 15 sec. For testing the reliability of hair preparation, hair samples from the participants were processed in duplicate. Following milling of hair segments, two 50 mg aliquots of powdered hair from a single hair segment were processed in parallel. Eighty microliters were removed from the vial and used for cortisol determination with a commercially available immunoassay with chemiluminescence detection (CLIA, IBL- Hamburg, Germany). The intraassay and interassay coefficient of variance of this assay is below 8%. The intra- and interassay coefficient of variation is less than 12% for hair cortisol concentrations between 15 and 100 pg/mg.

### Analytic Methods

Statistical analyses were conducted with STATA, version 10.0 (STATA Corp, College Station, TX) and the R Statistical Computing Language [20]. Ordinal Probit Models were used to assess the severity of participants' acute reactions. Negative Binomial Regression was used to assess ongoing distress levels, given that the distress measure was positive, integer-valued and skewed. Ordinary Least Squares was used for the continuous cortisol levels. Missing data due to partial survey non-response was handled using multiple imputation. Information from partially answered components of aggregate variables (ongoing distress, prior adversity) was included via observation level-priors and deterministic bounding [21].

Our complete model included: Gender, number of peers known personally, prior depression or anxiety diagnoses, prior interpersonal trauma, prior bereavements, prior other adverse events, number of social supports, and number of hours of media exposure to the deaths. For the severity of acute response and ongoing distress outcomes, all possible subset models using the software designed by Calcagno and Mazancourt [22] were run. Each subset was ranked by its Akaike Information Criterion (AIC), a goodness of fit measure based on the log-likelihood that penalizes for complexity. This has the advantage of allowing the presentation of parsimonious models, but also allows presentation of the best subset model summarized across all possible models. In the next section we review our main findings and discuss implications. Additional results, methodological details and visualizations are available in the Supplemental Appendix available through the third author's Dataverse website at http://hdl.handle.net/1902.1/22068.

## Results

### Sample and descriptive statistics

There were 134 surveys recorded online. We excluded individuals who did not complete any questions beyond the initial demographic items (n = 7) and one individual with a medical condition impacting the neuroendocrine system. Four individuals were over age 30 and were not undergraduate students (e.g., faculty and staff). We excluded these individuals given possible differences in psychological and physiological developmental stage and the number of years for which they were exposed to prior trauma experiences. The remaining sample (N = 122) is comprised of undergraduate students who ranged in age from 18–23 years (M = 20.13, SD = 1.14); 61.48% were female. The majority of participants were Caucasian (64.8%), followed by Asian/Pacific Islander (18.0%), Latino/Hispanic (6.6%), Black/African American (4.9%), Mixed Race (2.5%), and Other (3.3%). For reported regressions it was necessary to remove thirteen additional students who submitted incomplete surveys where all key variables of loss, distress, and prior trauma indicators were missing. This left N = 109 observations with limited partial missingness which was handled using multiple imputation, as described in the Supplemental Appendix. On average, students reported ‘knowing personally’ between one and two students who died; no one reported knowing no one who died (range 1–4, M 1.44; SD 0.72). Students reported spending on average 2.3 (SD 2.12) hours reading newspapers, web articles, or listening to radio or TV coverage of the deaths (range 0–11). Individuals reported experiencing between 0 to 6 interpersonal traumas, 0 to 3 prior bereavements (no participants who returned cortisol samples reported more than 2 prior bereavement experiences), and 0 to 9 prior other adversities. Both males and females reported a large network of individuals they felt they could turn to for emotional support (range 0–25, M 7.36, SD 4.97). Only 8% sought psychological or medical support (e.g., crisis hotline, psychologist, physician) available on campus to help students in the aftermath of the deaths.

Twenty-four individuals returned hair samples sufficient to analyze prior 3-month cortisol. Although some individuals provided longer hair samples, we focused on the 3-month values, in line with prior research [18] and to avoid bias against shorthaired people. Table 1 provides a description of the differences between participants who did and did not return a hair sample.

**Table 1:** Descriptive Statistics for all Study Variables by Participant Status^a^ (N = 122).

Characteristic^b^	Cortisol Sample^c^	No Cortisol Sample
	n = 24	n = 28
***Demographics***		
Gender (% female)	70.80	59.20
Age	20.25 (sd = 1.19)	20.10 (sd = 1.13)
***Ethnicity*** (%)		
Asian/Pacific Islander	20.80	16.30
Black/African American	0.00	6.12
Caucasian/White	75.00	62.20
Latino/Hispanic	4.17	7.14
Mixed Race	0.00	3.06
Other	0.00	5.10
Total	100.00	100.00
***Type of Adversity*** (mean)		
Prior Bereavement Events	0.96 (sd = .71)	1.10 (sd = 0.86)
Prior Other Adversity	1.22 (sd = 1.22)	1.56 (sd = 1.64)
Prior Interpersonal Trauma	0.08 (sd = 0.28)	0.58 (sd = 1.12)*
***Outcome Variables*** (mean)		
Ongoing Distress (range: 0–55)	7.36 (sd = 7.29)	11.42 (sd = 10.78)*
Severity of Acute Reaction to Deaths^d^	2.96 (sd = .955)	3.27 (sd = .876)
***Predictor Variables***		
% Prior Diagnosis of Depression	4.2	10.8
% Prior Diagnosis of Anxiety	4.7	8.3
# of Deceased Known Personally	1.38	1.42
Hours of Media Exposure to Deaths	2.31 (sd = 2.14)	2.04 (sd = 2.11)
# of Social Supports (range: 0–25)	7.38 (sd = 4.96)	7.29 (sd = 5.01)
% Using Support Resources^e^	4.2	10.8

*Notes*.

a Percentages may not total 100 due to rounding.

b Chi square analyses were conducted on dichotomous and categorical variables; unpaired t-tests were conducted on count and continuous variables.

c Number of participants who returned a usable hair sample.

d Mean score is given based on a scale from 1 to 5 with 1 being the least severe reaction and 5 being the most severe. No significant difference under a Fisher exact test of category independence, as well as with unpaired t-test.

e Support resources included individual attention from a mental health provider, a primary care provider, another kind of health professional, a crisis or support line, or a support group.

* *p*<.05.

### Predictors of distress

Women reported more severe acute reactions to the peer deaths (M 2.45, SD 0.87) than men (M 1.79, SD 0.90), t(111) = 4.06, *p*<0.001. Ordered Probit regression models (Table 2) revealed that after adjusting for all covariates, gender, the number of peers a student knew personally, and the number of hours of media exposure were positively associated with the severity of an acute reaction to the deaths (*p*'s<.05). Effect sizes are presented as Risk Ratios (RRs) for ‘best model’ variables. Women were 13.7 times more likely than men to report a more severe acute reaction. Participants who knew two deceased peers were approximately 3 times more likely to have an extreme reaction than those knowing one peer (RR = 3.53, 95% CI 1.6 to 7.25), while increasing media exposure from 2 hours (median) to 3 hours approximately doubled the likelihood of an extreme reaction (RR 1.73, 95% CI 1.31 to 2.38).

**Table 2:** Ordinal Probit Models Examining Predictors of Severity of Acute Reaction to the Deaths (N = 122).

	Model 1	Model 2	Effect Size^c^
	Full Model^a^	Best Subset^b^	“Extreme” Acute Reaction
	Coef (SE) (CI)	Coef (SE) (CI)	RR (CI)
Gender	0.98 (0.24)*, (0.50, 1.46)	.89 (0.23)* (0.44, 1.35)	13.7 (2.84, 45.6)
*Predictor variables*			
Prior Bereavement	0.01 (0.14), (−0.26, 0.28)		
Prior Interpersonal Trauma	0.06 (0.11), (−0.16, 0.28)		
Prior Other Adversity	−0.01 (0.07), (−0.15, 0.14)		
Prior Depression	0.29 (0.42), (−0.53, 1.12)		
Prior Anxiety	−0.85 (0.56), (−1.93, 0.24)		
# of Social Supports	0.03 (0.02), (−0.02, −0.07)		
# Deceased Known Personally	0.58 (0.17)** (0.26, 0.91)	0.54 (0.16)* (0.23, 0.85)	3.53 (1.6, 7.25)
Hours of Media Exposure	0.27 (0.06)** (0.15, 0.39)	0.25 (0.06)* (0.13, 0.36)	1.73 (1.31, 2.38)

*Notes*.

** *p*<.01,

* *p*<.05.

Gender was coded 0 = male, 1 = female.

a Full ordinal probit model containing all variables.

b Best performing subset of all models (including only the model-averaged important terms).

c Risk Ratio (RR) is a measure of effect size indicating the relative probability of being in the outcome category (i.e., having an “extreme” acute reaction to peer loss) based on different values of the independent variable (i.e., female gender, knowing more than one deceased peer, increasing from 2 to 3 hours of media exposure).

SE = Standard Error, CI = Confidence Interval.

Women reported significantly higher levels of ongoing distress (M 12.61, SD 11.38) than men (M 7.43, SD 7.32) (t(107) = 2.88, *p*<.01). Negative Binomial regression models (Table 3) revealed that, adjusting for all covariates, prior interpersonal trauma, diagnosis of depression, and fewer social supports were associated with ongoing distress (*p*'s<.05). Effect size estimates are presented as standard deviation shifts for ‘best model’ variables. A diagnosis of depression was associated with an 8.24-point increase in ongoing distress. An increase from 0 to 1 interpersonal trauma was associated with a 2.31-point increase in ongoing distress. Increasing social supports from 5 to 10 people was associated with a 2.31-point reduction in ongoing distress.

**Table 3:** Negative Binomial Models Examining Predictors of Ongoing Distress (BSI-18) (N = 122).

	Model 1	Model 2	Effect Size^c^ (CI)	Effect Size (CI)
	Full Model^a^	Best Subset^b^		
	Coef (SE), (CI)	Coef (SE), (CI)	1 SD Shift^d^	Min to Max Shift^e^
Gender	−0.03 (0.18), (−0.38, 0.33)			
*Predictor variables*				
Prior Bereavement	0.12 (0.09), (−0.07, 0.30)			
Prior Interpersonal Trauma	0.21 (0.07)*, (0.06, 0.36)	0.25 (0.07)**, (0.11, 0.4)	2.31 (0.97, 3.74)	31.9 (7.75, 75.64)
Prior Other Adversity	0.05 (0.05), (−0.05, 0.15)			
Prior Depression	0.61 (0.29)*, (0.05, 1.18)	0.62 (0.26)*, (0.1, 1.14)	—	8.24 (0.94, 18.4)
Prior Anxiety	−0.18 (0.38), (−0.94, 0.57)			
Severity of Acute Reaction	0.15 (0.10), (−0.05, 0.36)			
# of Social Supports	−0.05 (0.02)*, (−0.08, −0.01)	−0.05 (0.02)**, (−0.09, −0.02)	−2.31 (−3.88, −0.78)	−9.48 (−15.12, −3.59)
# Deceased Known Personally	0.03 (0.11), (−0.20, 0.25)			
Hours of Media Exposure	0.07 (0.04), (−0.01, 0.14)	0.09 (0.04)*, (0.02, 0.16)	0.80 (0.14, 1.49)	12.6 (1.64, 28.97)

*Notes*.

** *p*<.01,

* *p*<.05.

Gender was coded 0 = male, 1 = female.

a Full negative binomial model.

b Best performing subset of all models.

c Effect Size estimates are standard deviation shifts from the median, and minimum to max movements. These can be interpreted as standardized regression coefficients in the former case, and the maximal change in distress level attributable to the variable in the latter case.

d 1 SD Shift indicates the expected change in the distress score associated with a one standard deviation increase from the median of the independent variable.

e Min to Max shows the expected increase in distress associated with a move from the minimum to the independent variable to the maximum. Depression is a binary variable so we only show its min to max value. Interpersonal trauma reports findings for a shift from 0 to 1 and 0 to 6, respectively. Number of social supports reports the shift from 5 to 10 supports and 0 to 25 supports, respectively. Media exposure reports the shift from 2 to 3 hours and 0 to 11 hours, respectively.

SE = Standard Error, CI = Confidence Interval, SD = Standard Deviation.

### Cortisol Responses

Cortisol values in participants' hair samples ranged from 6.21 pg/mg to 50.1 pg/mg. Average cortisol values differed by ethnicity. Asian students' cortisol levels ranged from 16.7 to 37.5 (mean = 27.4). Caucasian students' cortisol levels ranged from 6.21 to 50.1 (mean = 22.37). The one Latin American individual's cortisol was 12.6 pg/mg. A larger sample size is necessary to determine if these are meaningful differences.

**Figure 1:**
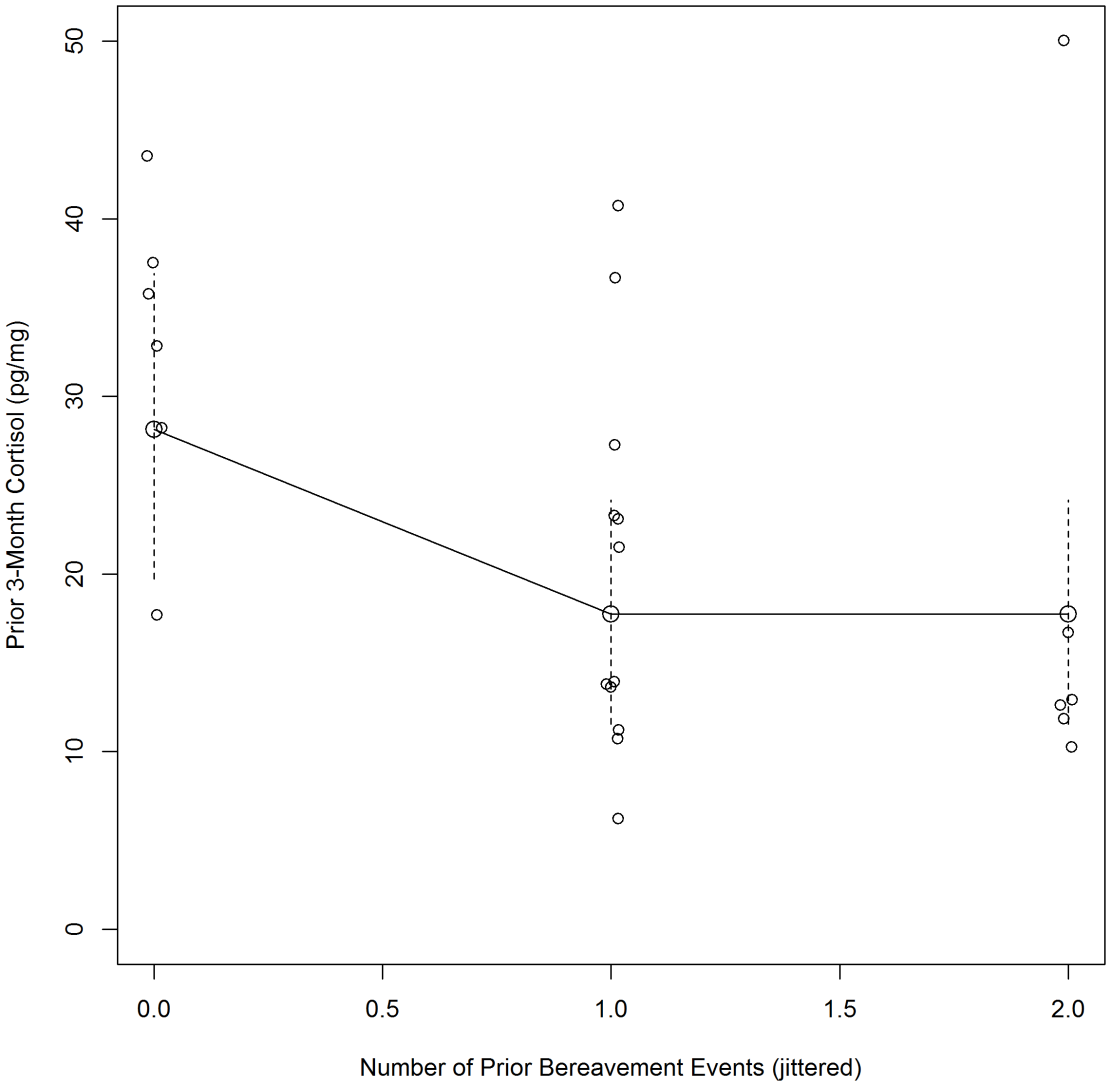
Prior Bereavement Events and Hair Cortisol During and Immediately Following the Period of Peer Deaths. Cortisol is plotted against prior bereavement events (jittered for legibility) along with the estimated effect of prior bereavement events. Solid lines connect the expected value of cortisol conditional on the number of prior bereavement events holding other covariates constant at their median (dashed lines indicate 95% confidence intervals around the expectation).

An Ordinary Least Squares Regression model (Table 4) revealed that none of the variables were significant (*p*'s>.05) when adjusting for all covariates in the full model (Model 1). However, in the best fitting model, prior bereavement experiences (e.g., death of friend or family member) were significantly associated with hair cortisol level (*p*<.05) while adjusting for female gender, number of peers known, number of social supports and media exposure. We explored this relationship more closely (see Figure 1). A negative relationship was seen between the number of prior bereavement experiences and cortisol levels during the period of peer deaths (*p*<.05). All but one (83%) of the individuals who had never suffered a prior bereavement showed high cortisol levels (>25 pg/mg), while only 22% of those who had experienced one or two prior losses had higher than average cortisol values. The effect associated with having had one versus no prior bereavement experiences is an expected 10 pg/mg point reduction in cortisol from the average of the ‘no loss’ group to the average of the ‘one prior bereavement’ group (−10.36, 95% CI −19.55, −1.18).

**Table 4:** Ordinary Least Squares Regression Models Examining Predictors of Cortisol Responses (N = 122).

	Model 1	Model 2
	Full Model^a^	Best Subset^b^
	Coef (SE), (CI)	Coef (SE), (CI)
Gender	9.60 (5.21)∧, (−0.61,19.81)	8.44 (4.29)∧, (0.03, 16.85)
*Predictor variables*		
Prior Bereavement	−11.05 (5.57)∧, (−21.97, −0.13)	−10.36 (4.69)*, (−19.55, −1.18)
Prior Interpersonal Trauma	3.34 (9.39), (−15.07, 21.74)	
Prior Other Adversity	1.23 (2.07), (−2.84, 05.29)	
Prior Depression	−0.86 (11.54), (−23.48, 21.75)	
Prior Anxiety	−4.47 (8.11), (−20.36, 11.43)	
# of Social Supports	0.84 (0.50), (−0.14, 1.82)	0.90 (0.43)∧, (0.05, 1.76)
# of Deceased Known personally	6.44 (3.77), (−0.95, 13.82)	5.67 (3.2)∧, (−0.61, 11.94)
Hours of Media Exposure	1.13 (1.37), (−1.56, 3.81)	1.73 (1.01), (−0.26, 3.71)

*Note*.

∧ *p*<.1,

* *p*<.05.

Gender was coded 0 = male, 1 = female.

a Full Ordinary Least Squares model containing all variables.

b Best performing subset of all models (including only the model-averaged important terms).

SE = Standard Error. CI = Confidence Interval.

## Discussion

Our findings support prior research on collective loss and extend knowledge about the psychological and physiological impact of exposure to sudden, repeated, unexpected peer deaths. Our results indicate that direct exposure (i.e., knowing more than two of the students who died) tripled the risk of a severe acute reaction to the deaths and indirect exposure (i.e., >3 hours of media exposure to the deaths) nearly doubled the risk. This is consistent with recent research showing that indirect exposure to collective trauma through the media is associated with psychological distress over time, similar to direct exposure, with a significant impact on health and wellbeing [2], [23].

Researchers have also found that prior trauma may exacerbate future responses to stress [8], [10]. We found that both the type and number of prior adversities (i.e., interpersonal trauma) were associated with ongoing distress responses following repeated loss. Specifically, those who had one prior interpersonal trauma versus none reported over a 2-point increase in distress in the months following the last peer death. For those who experienced a high number (e.g., 6) of prior interpersonal traumas, there was a near 32-point increase in ongoing distress (see Table 3). Our findings are consistent with research showing that female gender and prior depression are associated with elevated distress responses following trauma exposure [24]. In our sample, females were significantly more likely (13.7 times) to report a severe acute reaction to the peer deaths (even though 12 of the 14 deaths were males). A prior diagnosis of depression was also associated with an increase in distress and may be a clinically relevant consideration when predicting distress responses following repeated loss experiences. Social support appeared to mitigate ongoing distress. Our data suggest that having a very large network of friends to turn to for emotional support (25 individuals compared to 0) may be associated with a substantial (9.5-point) reduction in ongoing distress during and following a period of collective loss.

Our findings on the physiological impact of exposure to repeated, unexpected peer loss revealed that the single most important predictor of a cortisol response was whether or not a student had previously experienced the loss of a friend or family member. Although reports of distress were not associated with a physiological stress response (i.e., cortisol) in this sample, prior a review of research in this area [15] has highlighted equivocal results from studies on self-reported psychosocial distress and hair cortisol levels [25], [26]. Researchers who have found an association between self-reported distress and hair cortisol typically examined clinical samples (e.g., chronic pain patients, pregnant women), which may introduce complexities in stress-related physiological regulation that are not apparent in non-clinical samples [15]. Interestingly, Karlen and colleagues [27] found that while hair cortisol levels were not associated with self-reported distress among college students, they were associated with having experienced a serious life event (SLE) such as divorce or death of a close relative. In their study, students who reported a personal SLE in the past 3 months showed a twofold increase in hair cortisol levels [27].

We did find a negative relationship between the number of prior bereavement experiences and cortisol levels during the period of peer deaths. In fact, the majority of individuals who had never suffered a prior bereavement showed high cortisol levels compared to those who had experienced at least one prior loss. It may be the case that for individuals who had never experienced a prior bereavement, peer death was more likely to constitute an SLE. This finding supports the inoculation hypothesis in that individuals with some experience with prior bereavement maintained cortisol within average levels across the extended period of loss, while those with no prior experience displayed dysregulated cortisol levels. Seery and colleagues [11] found that individuals without a history of adversity showed greater functional impairment and distress following a collective national trauma than those with a moderate amount of prior adversities.

There are several limitations to this study. First, because the survey was fielded in the summer months, there was a lower response rate than might have been likely during the academic year. Second, only a portion of the respondents to our survey submitted useable hair samples. Finally, participants who returned hair samples reported fewer interpersonal traumas and less distress than those who did not. Given the few observations of cortisol responses available, we present these data as a preliminary investigation in need of replication.

Despite these limitations, we extend prior literature in several ways. We examined a topic that has not been well researched, namely an extended collective trauma of repeated peer loss. We applied a novel technique, hair cortisol analysis, to examine the physiological impact of this experience among young people (18 to 23 year olds). By doing so, we observed that prior bereavement experiences were associated with a type of HPA response to repeated peer loss. Previous research has shown that exposure to high levels of peer loss, particularly at a young age, is associated with chronic health conditions over the life span [28]. This study, albeit a preliminary step, may suggest one mechanism by which experiencing collective loss may contribute to physical health conditions via HPA dysregulation. Although research with larger samples is needed to replicate these findings, hair cortisol may be a promising biomarker for trauma researchers. The ability to assess retrospective HPA activity is especially important given the unpredictable nature of many traumas.

### Implications

We found that direct and indirect exposure to repeated loss was associated with ongoing distress several months following the tragedies. Elevated psychological distress, depression and the high pressure of an academic environment in combination are risk factors for poor health, functional impairment and even suicidal ideation for some teens [29]–[31]. Young adults who have not experienced bereavement or prior adversity may be at particular risk for health and adjustment concerns following the loss of peers. Taken together, these results support the need for campus wide intervention programs to meet student needs during and in the months following a collective tragedy.

This community tragedy involved a cluster of suicides (as well as accidents and illnesses) and the risk of contagion suicide was a key concern. Identifying students at risk for severe acute and prolonged distress was a priority. Although mental and physical health services and crisis outreach programs were made available to students, only a fraction of students who completed our survey actually utilized these resources. Peer social support may be a key outreach strategy; it can take place all over campus, reaching students who would otherwise not seek formal treatment. Peer support persons might be made aware of the possibility that prior interpersonal traumas may exacerbate distress levels when exposed to cumulative loss and address this in a sensitive manner, encouraging the use of therapeutic treatment options on campus. A network of peer support across campus may be particularly important to foster resilient responses to future stress and more rapidly identify students at risk for the negative effects of repeated loss.

These findings also have implications for individuals in occupations where exposure to peer death is highly likely (police, fire fighters). First responders and military soldiers who have never experienced peer death may be particularly vulnerable to losing peers for the first time, which may be compounded by the requirement to continue working under highly stressful conditions. These recommendations for peer support programs and identifying individuals who have not experienced prior loss can also be applied to other population groups in which peer loss is highly likely.
